# Exploring the Modes of Action of Phosphorus-Based Flame Retardants in Polymeric Systems

**DOI:** 10.3390/ma10050455

**Published:** 2017-04-26

**Authors:** Sebastian Rabe, Yuttapong Chuenban, Bernhard Schartel

**Affiliations:** Bundesanstalt für Materialforschung und -prüfung (BAM), 12205 Berlin, Germany; sebastian.rabe@bam.de (S.R.); yuttapong1131@hotmail.com (Y.C.)

**Keywords:** flame retardants, flame inhibition, cone calorimeter, aluminum diethyl phosphinate, polyester, PMMA, epoxy resin, red phosphorus, BDP

## Abstract

Phosphorus-based flame retardants were incorporated into different, easily preparable matrices, such as polymeric thermoset resins and paraffin as a proposed model for polyolefins and investigated for their flame retardancy performance. The favored mode of action of each flame retardant was identified in each respective system and at each respective concentration. Thermogravimetric analysis was used in combination with infrared spectroscopy of the evolved gas to determine the pyrolysis behavior, residue formation and the release of phosphorus species. Forced flaming tests in the cone calorimeter provided insight into burning behavior and macroscopic residue effects. The results were put into relation to the phosphorus content to reveal correlations between phosphorus concentration in the gas phase and flame inhibition performance, as well as phosphorus concentration in the residue and condensed phase activity. Total heat evolved (fire load) and peak heat release rate were calculated based on changes in the effective heat of combustion and residue, and then compared with the measured values to address the modes of action of the flame retardants quantitatively. The quantification of flame inhibition, charring, and the protective layer effect measure the non-linear flame retardancy effects as functions of the phosphorus concentration. Overall, this screening approach using easily preparable polymer systems provides great insight into the effect of phosphorus in different flame retarded polymers, with regard to polymer structure, phosphorus concentration, and phosphorus species.

## 1. Introduction

Nowadays, the ever growing numbers of different flame retardants for all kinds of applications, along with their variations in concentration, particle size distribution and so on, create a need for screening methods that allow time and material savings while yielding significant results and enabling reasonable conclusions. Several different approaches have been taken to accelerate fire testing, such as the microscale combustion calorimeter, the rapid cone calorimeter and the rapid mass calorimeter [1,2,3,4]. These methods are specifically designed for screening the fire performance of large numbers of samples. However, all of the methods require specific sample preparation, such as extrusion and injection molding, to ensure homogenous implementation of the flame retardant in the thermoplastic. With the acceleration of the actual testing method, the bottleneck shifts towards sample preparation. Thus, here a screening approach is presented that uses cone calorimeter investigations on different thermosets and on paraffin, which is proposed as a model for polyolefins, to address different phosphorous flame retardants in different concentrations.

Phosphorus-based flame retardants have been proposed to replace halogenated flame retarding additives due to their good fire properties and better environmental sustainability [5,6,7]. They exhibit all kinds of flame retardant modes of action, such as flame inhibition in the gas phase and char enhancement, and intumescence and formation of inorganic glass in the condensed phase [6,8,9,10,11,12,13,14,15,16]. In many polymers, aluminum diethyl phosphinate (AlPi) is reported to exhibit high activity in the gas phase by releasing diethylphosphinic acid [17,18,19,20]. Often only a small fraction of it remains in the condensed phase to induce some char or residue formation [21]. Phosphate-based flame retardants are reported to act in the condensed phase as acid precursors, leading to char formation by esterification and dehydration [22,23,24]. Nevertheless, if the phosphate esters are released into the gas phase instead of reacting with the decomposing polymer, they show high flame inhibiting effects [25,26]. Thus, phosphate flame retardants such as bisphenol-A bis(diphenyl phosphate) (BDP), triphenyl phosphate (TPP) and resorcinol bis(diphenyl phosphate) (RDP) have different flame inhibition effectiveness, due to variations in decomposition behavior and releasability [25,27]. Despite its flammability hazard, red phosphorus is used as a flame retardant as well. It can act in both the gas phase and the condensed phase [8,16,28]. Indeed, phosphorous flame retardants often act in the condensed phase and in the gas phase at the same time [8,10,26,29]. It was also demonstrated that the existence and efficiency of the mode of action as well as its effect on the performance of the flame retardant depends not only on its chemical structure, but also strongly on its interaction with the polymeric matrix in which it is incorporated [8,11,15,26,30].

In this work, two AlPi flame retardants with different particle size distributions were used, as well as BDP and red phosphorus. Thus, it was possible to investigate and compare a range of different phosphorus sources with different phosphorus content and particle sizes. To achieve easier and faster sample preparation, the flame retardants were incorporated into resins, which are cured under laboratory conditions and into paraffin. Here, epoxy-, polyester- and acrylate-based resins were used by simply mixing them with the additive and molding in small amounts in the laboratory. It has to be noted, however, that the used epoxy resin cannot be seen as an example for all epoxy resins, since burning behavior and behavior of the flame retardants in the respective resin may be different. The group of polyolefins was replaced by paraffin. The relatively fast compounding aims to shift the bottleneck in the testing process away from sample preparation. Furthermore, the results obtained by means of these easily compounded polymeric systems are explained using general model assumptions.

## 2. Materials and Methods

### 2.1. Thermogravimetric Analysis Coupled with FTIR

Thermogravimetric analysis was conducted on a TG 209 F1 Iris (Netzsch Instruments, Selb, Germany). Specimens were provided in 5 mg portions of powder in a ceramic crucible, which were pyrolyzed under nitrogen at a heating rate of 10 K/min. Pyrolysis was performed in the absence of oxygen to simulate the oxygen-lean conditions in a laminar flame. To analyze and identify the gases during pyrolysis, the TGA was coupled via a transfer line with a Tensor 27 infrared spectrometer (Bruker Optics, Ettlingen, Germany). The gas cell and transfer line were operated at a temperature of 270 °C to ensure transport of the pyrolysis gases without condensation effects [31].

### 2.2. Cone Calorimeter

Forced flaming combustion tests were performed using a cone calorimeter (FTT, East Grinstead, UK) in accordance with ISO 5660. Specimens 100 × 100 × 3 mm^3^ in size were conditioned for 48 h at 23 °C and 50% relative humidity, wrapped in an aluminum tray, and irradiated with 50 kW/m^2^ at a distance of 35 mm from the cone heater [32] to ensure sufficient spacing for the greatest possible residue formation. Samples were measured in triplicate when the first two measurements deviated by more than 10% in any result.

### 2.3. Elemental Analysis

Elemental analysis was performed by Mikroanalytisches Laboratorium Kolbe (Mühlheim an der Ruhr, Germany).

### 2.4. Materials

#### 2.4.1. Flame Retardants

Aluminum diethylphosphinate (AlPi) was used in two different varieties, Exolit OP935 and Exolit OP1230 (Clariant). Exolit OP935 is a fine-grained AlPi developed especially for adhesive applications, and is established as an effective flame inhibitor in epoxy, acrylic and TPE resins [18,33]. The highly stable Exolit OP 1230 is widely used for high-temperature polyamides, as well as in polyamide-AlPi mixtures [34,35,36]. Both flame retardants contain around 23.5 wt % phosphorus. Red phosphorus (Exolit RP607, Clariant) is typically used in thermoplastics for electronics applications [28]. As it is the most concentrated source of phosphorus as a flame retardant, its application concentration generally lies below 10 wt %, and it is usually applied as encapsulated material. Bisphenol-A bis(diphenyl phosphate) (BDP) is an oligomeric aryl phosphate ester designed for use in engineering resins such as PC/ABS [5,6]. Since BDP contains only around 9 wt % of phosphorus, a higher BDP load must be considered in the material. BDP was used in concentrations of 10, 20, 25 and 35 wt % to reach the effective range of phosphorus concentration. All other investigated flame retardants were concentrated at 5, 10, 15 and 20 wt %.

#### 2.4.2. Resin Systems

Epoxy resins have become important materials for electronics and lightweight construction, especially as fiber reinforced composites [37,38]. Bisphenol-A diglycidyl ether (DGEBA, Araldit MY740, Bodo Möller Chemie GmbH, Offenbach, Germany) and isophorone diamine (IPDA, Merck KGaA, Darmstadt, Germany) was used as an example for an epoxy resin [39]. IPDA and the respective flame retardant were added to DGEBA and stirred until a homogenous mixture was obtained. Forty grams of the mixture was poured into a 100 × 100 × 6 mm³ aluminum mold and cured for 30 min at 80 °C and for 70 min at 120 °C. The aluminum frame was stabilized with weights to prevent deformation during curing. The edges of the finished specimens had to be ground down in order to obtain a flat surface. The rest of the mixture was also cured as described above, followed by grinding to obtain powder for TGA-FTIR measurements.

As an acrylate thermoset, a pre-accelerated PMMA resin with an incorporated, UV-induced curing agent was used (S. u. K. Hock GmbH, Regen, Germany). The respective flame retardant was added to the methyl methacrylate resin and stirred until homogeneity was achieved. The mixture was then poured into aluminum molds and cured at room temperature and irradiated by a daylight spectrum lamp for 12 h. Systems with red phosphorus could not be produced because the curing process was hindered so that complete hardening could not be achieved.

For preparation of the polyester system, the flame retardant was added to a mixture of polyester resin L800 and the curing agent methyl ethyl ketone peroxide (MEKP) (S. u. K. Hock GmbH, Regen, Germany). The mixture was then poured into aluminum molds and cured at room temperature for 8 h. The edges of the molds were also weighed down to prevent deformation during curing. Red phosphorus prevented the resin from curing, so that a polyester formulation with RP as flame retardant could not be manufactured.

To represent the group of polyolefins, paraffin pellets were melted at 75 °C and combined with the flame retardant. The hot mixture was poured into the aluminum molds and cooled down to room temperature. It was not possible to implement BDP into paraffin because the two phases were immiscible. BDP accumulated as a bubble underneath the paraffin in the mold. All produced materials were given a label according to Table 1.

## 3. Results and Discussion

### 3.1. Thermogravimetric Analysis Results

**Figure 1:**
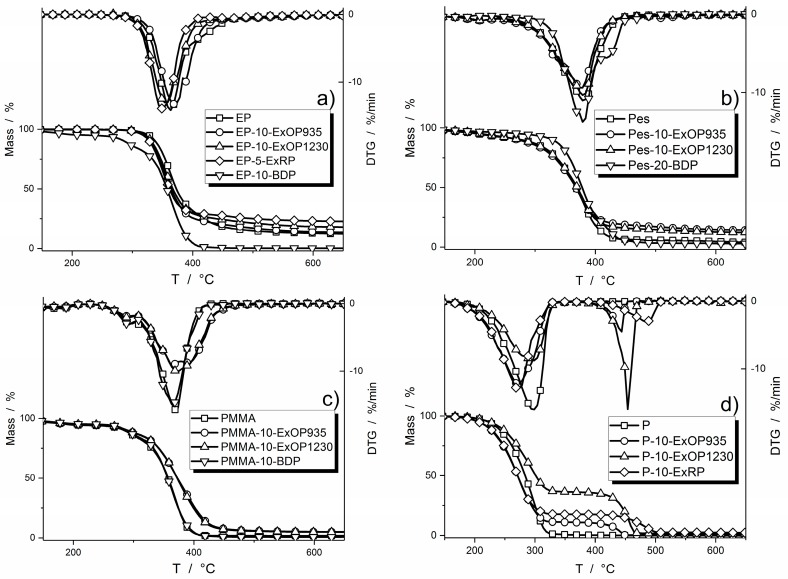
Thermogravimetric (TG) and derivative thermogravimetric (DTG) results for selected flame retarded and non-flame retarded specimens with: epoxy resin (**a**); polyester resin (**b**); PMMA resin (**c**); and paraffin (**d**) as polymer matrix.

All investigated systems, with and without flame retardant, were investigated via thermogravimetric analysis in the absence of oxygen. Mass and DTG curves are displayed in Figure 1 and results are listed in Table 2.

The start of the decomposition T_5% ML_ (at which 5 wt % of the material was lost) for epoxy resin formulations shifted towards lower temperatures when flame retardants were added. EP-10-ExOP935 and EP-10-ExOP1230 showed decomposition behavior similar to the pure epoxy resin. The temperature of the maximum mass loss rate, T_DTGmax_, was shifted to temperatures 5 and 7 °C lower, respectively. With the non-flame retarded epoxy resin producing 10.7 wt % of residue, replacement of 10 wt % of the resin with AlPi flame retardants would still produce 9.6 wt % residue. Since residue formation increased to 12.1 wt %, 2.5 wt % of the residue results from the incorporation of the flame retardant. Epoxy resin with 5 wt % of red phosphorus flame retardant added began decomposition at an even lower temperature, exhibiting a lower T_DTGmax_, and an increase in residue of around 6.8 wt %. This means that carbonaceous char was produced. Besides the drastic lowering of the temperature at the start of decomposition, incorporation of 10 wt % of BDP in the epoxy resin resulted in a second decomposition step prior to the main one. It also formed much less residue than the other epoxy formulations, leading to the conclusion that this amount of BDP prevents residue production in epoxy resin. The temperature of maximum decomposition was shifted to temperatures 5 °C higher.

For the polyester system, the beginning of decomposition shifted to temperatures only slightly higher for Pes-10-ExOP935 and Pes-10-ExOP1230, but the effect was stronger when BDP was added. Ten weight percent of Exolit OP935 promoted the production of residue, increasing the amount by 11.4 wt %. Carbonaceous char was formed. For 10 wt % of Exolit OP1230 in polyester resin, the residue formation was promoted by 9.1 wt % compared to the non-flame retarded polyester resin. Pes-20-BDP produced only 0.3 wt % more residue. The temperatures of maximum decomposition did not alter much with the addition of a respective flame retardant.

In the PMMA resin, the starting temperature for decomposition shifted to higher temperatures with the addition of flame retardants. Incorporation of 10 wt % Exolit OP935 or OP1230 increased the temperature of 5 wt % mass loss by around 37 °C, whereas BDP raised it by more than 50 °C. There are no differences in decomposition observed between Exolit OP935 and OP1230. Ten weight percent of either flame retardant in PMMA resin increased the amount of residue by 3 wt %, consisting of inorganic ash. The amount of residue of PMMA-10-BDP was insignificant. Only for 10% BDP was a relevant shift of T_DTGmax_ by 6% to lower temperatures observed.

For paraffin, all of the flame retarded formulations showed a two-step decomposition process, clearly visible in both the mass loss and the mass loss rate curves (Figure 1d). The AlPi flame retardant Exolit OP935 as well as the red phosphorus caused a decrease in the temperature at which decomposition begins. Furthermore, both flame retardants caused a shift in the maximum of the mass loss rate to temperatures 22 to 23% lower. In the DTG curves it becomes clear that the formulation with 10 wt % of Exolit OP1230 showed the highest mass loss rate in the second step of decomposition. It was even higher than what is considered the main decomposition step for the other formulations, changing the T_DTGmax_ to 454 °C. The residues produced by all paraffin formulations are negligible. These results clarified that the phosphorus-based flame retardants only slightly promote the formation of a residue, especially at relatively low concentrations of up to 10 wt %, and thus are mainly released into the gas phase. The varied particle size distribution in Exolit OP935 and Exolit OP1230 had no significant influence on the thermal decomposition properties.

### 3.2. FTIR Spectra of the Gaseous Phase

**Figure 2:**
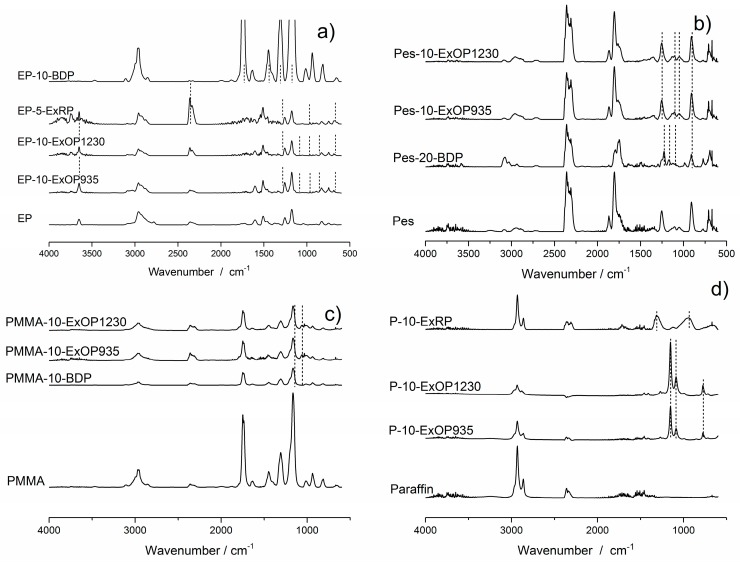
**Fourier transform infrared** (FTIR) spectra at the respective main decomposition step of flame retardants in: epoxy (**a**); polyester (**b**); PMMA (**c**); and paraffin (**d**) systems. Dotted lines were included as an aid to highlight smaller peaks from phosphorus species.

To confirm that phosphorus species were released into the gas phase, the pyrolysis gases evolved from TGA measurements were introduced into an IR cell. It was possible to identify various volatile phosphorus species. The FTIR spectra of the investigated formulations are shown in Figure 2. These experiments simply serve as a way to enable a discussion about the gas phase activity due to the release of phosphorus species.

The AlPi flame retardants Exolit OP935 and OP1230 showed peaks at wavenumbers 854, 3650 and 1265 cm^−1^ in the epoxy resin, indicating the P–O, P–OH and P=O-stretch of diethylphosphinic acid. At 1078 cm^−1^, a weak peak expressed the PO^2−^ anion of the AlPi molecule, whereas the peak at 952 cm^−1^ signified the production of ethene by a P–C bond break in AlPi. A phosphinate peak was found at 670 cm^−1^ [21,40,41]. Red phosphorus showed peaks at 670, 922, 1256 and 2375 cm^−1^, which are representative for PO_4_^3−^, P–OP, P=O and P–OH vibrations. In the EP-10-BDP formulation, several peaks corresponding to phenolic derivatives of bisphenol-A were found at 1261, 1173, 1490 and 1620 cm^−1^, which represent aromatic phosphate esters.

In the polyester resin, Exolit OP935 and 1230 showed peaks at 1105 and 1053 cm^−1^, which display PO^2−^ vibration. The peak at 1257 cm^−1^ is attributed to P=O. A peak for a P–O–P vibration was found at 910 cm^−1^. In BDP, those vibrations occurred at 1165, 1087, 1230 and 910 cm^−1^.

Peaks related to phosphorus species are less pronounced in the flame retarded PMMA resin formulations. The strong release of methyl methycrylate, verified by the characteristic peaks such as the C–H stretch at around 2950 cm^−1^, the C=O stretch at 1700 cm^−1^, the C=C stretch at 1600 cm^−1^ and the C–O stretch at around 1200 cm^−1^, concealed most of the phosphorus-related peaks. Only the peaks at 1056 and 1168 cm^−1^ suggest the existence of PO^2−^ species in the gas phase. However, Exolit OP935 and OP1230 incorporated into paraffin showed strong PO^2−^ peaks at 1156 and 1085 cm^−1^, as well as a peak at 775 cm^−1^ which is attributed to diethylphosphinic acid. Red phosphorus in paraffin did not exhibit phosphorus-related peaks at the first decomposition step. However, at the second decomposition step, phosphorus was released as mixtures of undefined low-oxidized phosphorus oxides indicated by broad peaks at around 1300 and 950 cm^−1^. Earlier work showed that phosphorus was released as P_4_ molecules, breaking down to P_2_ molecules with increasing temperature [16].

The results from the FTIR experiments demonstrated that phosphorus species are traceable in the gaseous phase and thus are released during pyrolysis of the formulations. This is the premise of the following investigations into the extent to which phosphorus compounds and the concentration of phosphorus in the gas phase are attributed to flame inhibition, and whether the remaining phosphorus in the residue has any effect on other modes of action in flame retardancy.

### 3.3. Forced Flaming Fire Behavior: Cone Calorimeter

#### 3.3.1. Comparison of Heat Release Rates

**Figure 3:**
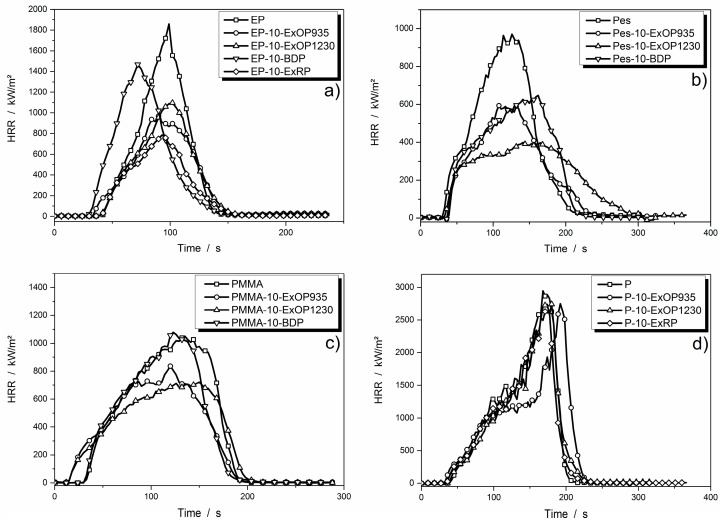
Cone calorimeter heat release rate (HRR) curves of flame retardants in: DGEBA/IPDA (**a**); Pes-resin (**b**); PMMA resin (**c**); and paraffin (**d**) with a concentration of 10 wt %.

Forced flaming fire tests were conducted in the cone calorimeter. At first, it was interesting to see the differences between the investigated flame retardants at the same concentration in different matrices. The heat release rate (HRR) curves are displayed in Figure 3. The results of all cone calorimeter measurements are summarized in Table 3.

Except for paraffin, the systems clearly showed improvements in terms of flame retardancy, such as reduction in peak heat release rate (PHRR) and total heat evolved (THE). In the epoxy resin, the formulation with 10 wt % of BDP was the least effective; it lowered the PHRR but shortened the time to ignition. Ten weight percent of Exolit OP935 and OP1230 further reduced PHRR, with OP935 showing slightly better performance. However, their efficiency at 10 wt % was similar. Compared to the residue analysis results from TGA, the increase in residue was much more evident in the cone calorimeter, with an increase of 10% for the highest investigated flame retardant load. Red phosphorus induced the largest reduction in HRR, although an even greater reduction was expected because of the high phosphorus content. This suggests that not only the phosphorus content of a flame retardant is responsible for its performance, but also the way the phosphorus is incorporated. Compared to the results from the thermogravimetric analysis, the residue produced is not as distinct, especially not for red phosphorus. At the same time, THE is reduced by almost 50% for EP-15-ExRP. This allows the conclusion that gas phase activity is mainly responsible for the high THE reduction. However, the residue produced by BDP increased strongly with higher flame retardant load. This suggests that there is a clear tendency for condensed phase activity of BDP. In the epoxy resin, 15 wt % of red phosphorus as well as 35 wt % of BDP showed the best HRR and THE reduction.

Polyester resin showed a PHRR of 900 to 1000 kW/m^2^ without any flame retardant. The flame retardants Exolit OP935 and BDP caused a similar reduction in PHRR to around 600 kW/m^2^, even though BDP has lower phosphorus content. The performance of 10 wt % Exolit OP1230 in polyester resin exceeded those of the other polyester formulations, as the PHRR was reduced further, to around 400 kW/m^2^. In the PMMA resin, the formulation with 10 wt % of BDP had about the same performance as the non-flame retarded resin. Residue formation of the polyester resin formulations did not show salience; amounts increased with increasing flame retardant load, but remained below 10 wt %. It was found that Exolit OP1230 resulted in the greatest HRR and THE reduction, especially at 20 wt %.

When Exolit OP935 or 1230 were incorporated into PMMA resin, the PHRR decreased but the time to ignition was shortened. Exolit OP1230 showed slightly lower PHRR but a longer time of burning. Residue formations were in great accordance with results from thermogravimetric analysis, and demonstrated that all flame retardants work mainly via gas phase activity. Incorporation of BDP did not deliver satisfying results in HRR and THE reduction and was thus considered the worst additive for these purposes.

Forced flaming heat release tests on paraffin and the respective flame retarded formulations did not differ substantially as a pure system or with 10 wt % flame retardant load. Only the system with 10 wt % of Exolit OP935 showed prolongation of the small plateau of steady burning and a slight shift of the PHRR to later times. Observation of the burning process of the formulations including red phosphorus showed a peculiarity—at the end of the burning process the flame became very bright and smoke production increased. Red phosphorus did not contribute significantly to inhibiting the burning process. Rather, it burned away at the end of the burning process. Reductions in PHRR were thus due to fuel replacement by the flame retardant. No significant residue was formed. Only at high concentrations of the AlPi flame retardants were 2 to 4 wt % of residue measured. However, it was observed that they formed towards the end of the burning, when the flame was about to extinguish; thus, they were not able to affect the burning behavior through condensed phase activity. Overall, 20 wt % of Exolit OP935 reduced PHRR from 2972 to 1130 kW/m^2^ and THE from 210 to 188 MJ/m^2^, and is thus considered to be the best suited additive in paraffin.

**Figure 4:**
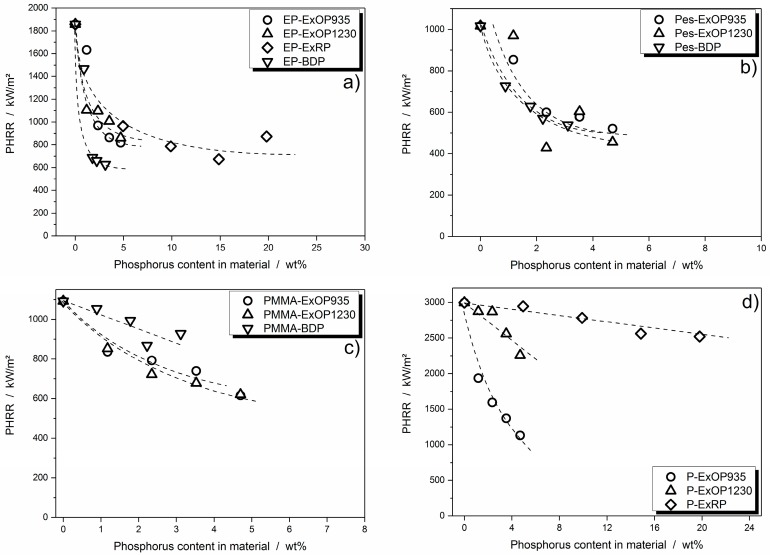
**Peak of heat release rate** (PHRR) of epoxy resin (**a**), polyester resin (**b**), PMMA resin (**c**) and paraffin (**d**) systems plotted against amount of phosphorus in the bulk material. The dotted lines are included as guides for the eye analogous to the findings from Brehme et al. for comparable materials [31].

One of the most important characteristics when it comes to the fire performance of a flame retardant in a polymeric system is the change in PHRR. The reduction in PHRR signifies several other phenomena: reduction in the effective heat of combustion, reduction in released fuel due to an increased amount of residue, and the effect of a protective layer are all reflected by this measure. To illustrate the correlation between PHRR reduction and phosphorus content in a flame retardant, they were plotted in Figure 4.

The DGEBA/IPDA epoxy resin showed a very pronounced leveling off of flame retardancy effectiveness for all investigated flame retardants. This leveling off set in at around 10 wt % phosphorus content for RP, whereas the AlPi flame retardants Exolit OP935 and OP1230 already displayed this effect at a lower phosphorus content. For BDP, which had the lowest phosphorus content of all investigated flame retardants, the reduction in PHRR was highest, but leveled off at around 700 to 600 kW/m^2^ at even low phosphorus concentrations (Figure 4a).

The leveling off of PHRR reduction effectiveness was also visible in the polyester resin. However, since the absolute reduction in PHRR was not as high as in the epoxy resin, the leveling off was not as pronounced. The PHRR reduction was more or less the same for all three incorporated flame retardants (Figure 4b). Deviations in the Exolit OP1230 polyester system may derive from slight variations in curing time.

Flame retardants in the PMMA resin tended to level off in effectiveness in terms of PHRR reduction. However, the decay in effectiveness was more gradual, especially since the pure reduction in PHRR was less significant than in the epoxy resin. BDP in the PMMA resin even seemed to have a linear decay in PHRR with increasing phosphorus content (Figure 4c).

Flame retardants in paraffin did not exhibit leveling off, but rather a linear decay in PHRR. Red phosphorus did not lead to a significant reduction in PHRR. Exolit OP935 was the most effective flame retardant, reducing the PHRR from 3000 to around 1000 kW/m^2^ with a phosphorus content of only 4.7 wt % (Figure 4d). It would now be interesting to establish how much of the flame retardant performance actually resulted from gas phase activity, namely flame inhibition.

#### 3.3.2. Flame Inhibition Dependency on Phosphorus Content Released into Gas Phase

**Figure 5:**
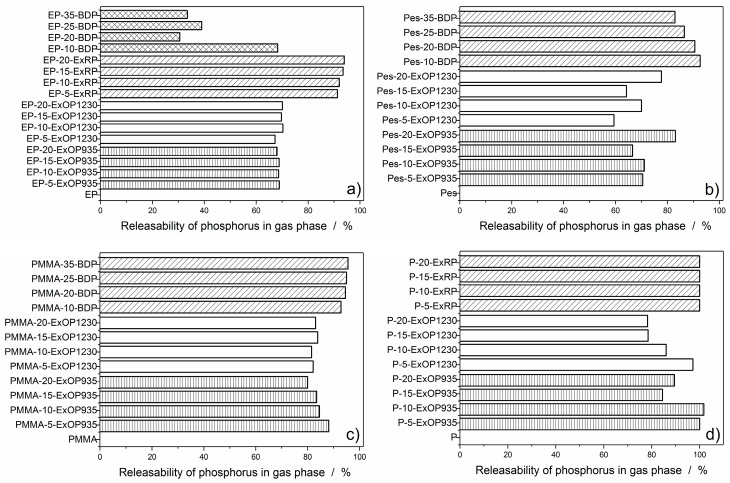
Bar graphs of the percentage of phosphorus released in the gas phase during combustion for epoxy resin (**a**); polyester resin (**b**); PMMA resin (**c**); and paraffin (**d**) formulations.

To investigate gas phase activity, it was first necessary to determine the amount of phosphorus released into the gas phase. This was calculated by subtracting the measured phosphorus content remaining in the residue from the total amount of phosphorus in the sample. Concentration of phosphorus inside the residue was measured via elemental analysis. For visualization purposes, phosphorus release is depicted in a bar graph for every formulation in Figure 5.

In the epoxy resin, there were no differences in phosphorus release between Exolit OP935 and OP1230. At any flame retardant load in the sample, around 70% of the phosphorus was released into the gas phase. Phosphorus release at 10 wt % flame retardant load was similar to that of the AlPi flame retardants at any concentration, but with increasing BDP load, this amount was significantly reduced to less than half of the phosphorus released into the gas phase. Red phosphorus released over 90% of its phosphorus into the gas phase and showed a slight increase with increasing flame retardant load (Figure 5a).

Exolit OP935 in polyester resin had a higher phosphorus release than Exolit OP1230. The highest release of phosphorus was also at the highest flame retardant load. By contrast, formulations with BDP had the highest phosphorus release at 10 wt % of BDP load, which gradually decreased to around 80% with increasing amounts of BDP (Figure 5b).

In the PMMA resin, Exolit OP935 showed the maximum phosphorus release at the lowest amount of flame retardant. This decreased with increasing flame retardant load. The phosphorus release of Exolit OP1230 was constant at around 80 to 85% at all investigated flame retardant loads. For BDP the phosphorus release was at a very high level, at around 90 to 95% with increasing load in the matrix (Figure 5c).

**Figure 6:**
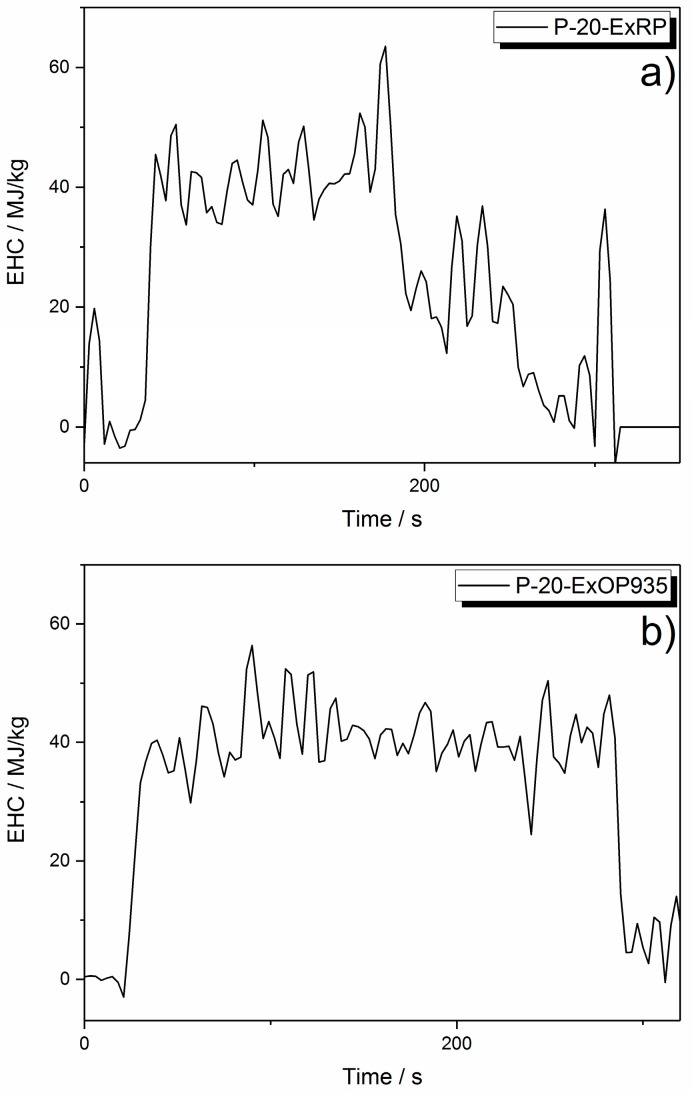
Effective heat of combustion: for P-20-ExRP exhibiting a two-part progression (**a**); and for P-20-ExOP935 as an example for a constant EHC (**b**).

Exolit OP935 and OP1230 in paraffin both showed decreasing phosphorus release with increasing flame retardant load. However, this effect was more significant with Exolit OP1230. The highest phosphorus release into the gas phase was at 10 wt % of flame retardant load. Formulations with RP left no residue at all; thus, all phosphorus was released into gas phase during burning (Figure 5d). It was observed that, towards the end of the burning process, the flame turned very bright. It is assumed that the red phosphorus was burning separately, resulting in this bright flame, and thus did not take part in any pyrolysis reaction with the matrix. This is supported by monitoring the effective heat of combustion (EHC) during the test. As seen in Figure 6, the EHC is split into two areas, the first one attributed to the burning of the paraffin matrix at around 40 MJ/kg. After reaching the PHRR at around 200 s, the EHC curve features a second, lower plateau at approximately 20 MJ/kg with a final decay, which is associated with the burning of red phosphorus. This is in analogy to the different burning phases observed for example in polymer blends [42]. Contrarily, the EHC of 20 wt % AlPi incorporated in paraffin features a constant EHC throughout the entire period of burning. This shows, that the decomposition temperatures of red phosphorus and the paraffin matrix were not overlapping and thus did not ensure an active flame retardancy.

**Figure 7:**
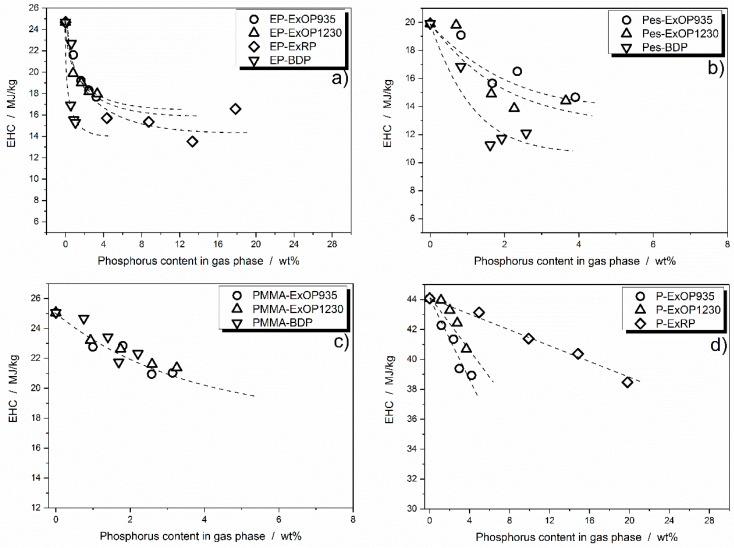
Effective heat of combustion plotted versus the actual amount of phosphorus released into the gas phase during combustion in: DGEBA/IPDA (**a**); polyester resin (**b**); PMMA resin (**c**); and paraffin (**d**).

Overall, it is demonstrated that phosphorus release is controlled by the polymer matrix, the phosphorus species itself and the concentration of phosphorus in the specimen. To learn about the way flame inhibition is influenced by the amount of phosphorus release into the gas phase, the effective heat of combustion (EHC) is plotted versus phosphorus concentration in the gas phase (Figure 7). The EHC measured by the cone calorimeter includes the effective heat of the combustion of the volatiles multiplied by the combustion efficiency of the flame, and thus is a measure for the efficiency of a flame retardant in terms of flame inhibition and gas phase activity in general.

**Figure 8:**
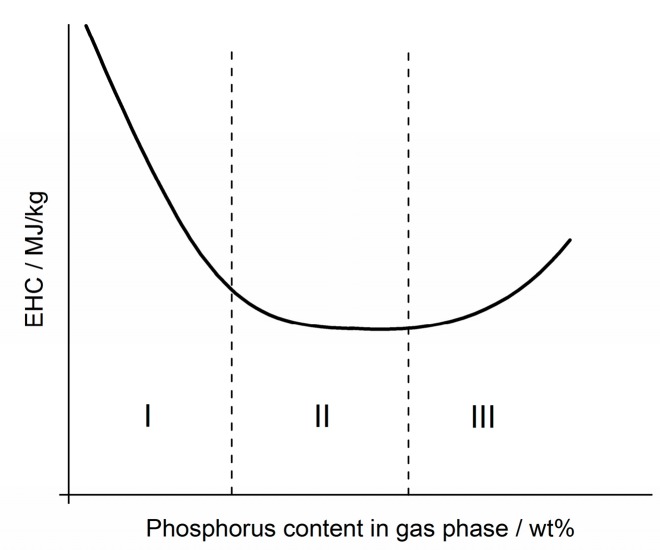
Proposed model for the dependency of effective heat of combustion on the phosphorus content released into the gas phase based on previous work by Brehme et al. [31]. The curve progression follows a linear decrease of EHC (I) and leads to a leveling off of EHC reduction (II). Further phosphorus increase is proposed to have a negative impact on efficiency, thus the increase in EHC (III).

As seen in Figure 7a especially, the curve progression of EHC levels off with increasing phosphorus content in the gas phase. The model curve in Figure 8 is proposed as a basis for the following discussion and is based on findings in previous works [31,43], in which the EHC first dwindles in a linear fashion (I), up to a certain level where the effectiveness in EHC reduction levels off (II). This effect is very prominent in epoxy resin, and less pronounced in the polyester system. The phase is described as (III) in Figure 8, which depicts an increase in EHC with even higher amounts of phosphorus in the gas phase. This converse effect occurs due to increased burning of the phosphorus species without exhibiting any flame inhibiting effect.

In epoxy resin, RP showed a higher reduction in EHC than the AlPi flame retardants Exolit OP935 and OP1230. More phosphorus was released into the gas phases, contributing strongly to EHC reduction. However, BDP displayed a similar EHC reduction while releasing less phosphorus into the gas phase, and the leveling off set in at around 15 MJ/kg. This demonstrated that not only the pure amount of phosphorus but also the nature of the phosphorus species had an influence on the flame inhibition [23]. At higher concentrations, all formulations showed a leveling off of the EHC reduction in the same range (Figure 7a). EHC was reduced by around 55–65% before an increase in phosphorus content in the gas phase ceased to have a beneficial effect.

In the polyester resin, BDP showed higher efficiency than Exolit OP935 and OP1230, reducing the EHC to around 11 MJ/kg (Figure 7b). Nevertheless, in this system, too, it became clear that, with increasing phosphorus concentration in the gas phase, the reduction in EHC, i.e., the effectiveness of a flame retardant in terms of gas phase activity, reached its limit. For the polyester resin, the minimum EHC was found to be at around 50 to 65% of the non-flame retarded formulation, depending on which flame retardant is used.

In contrast, formulations with PMMA resin as the polymer matrix showed no clear differences in EHC reduction behavior, and a slight tendency toward leveling off is possible (Figure 7c).

Paraffin differed from the other investigated systems in that no leveling off in EHC reduction was apparent. All three of the flame retardants used exhibited approximately linear behavior with increased concentrations in the gas phase, as also observed for the decrease in PHRR with increasing phosphorus load (Figure 7d). From these investigations, it can be concluded that, for the two systems epoxy resin and polyester resin, there is a maximum concentration of phosphorus at which flame inhibition has its highest efficiency as a mode of action. After that point, a higher load of flame retardant and thus phosphorus concentration will more likely contribute to condensed phase activity. Furthermore, the polymeric matrix in which the flame retardants are used can alter their efficiency significantly. The PMMA systems showed a slight tendency towards a non-linear EHC reduction with increasing phosphorus content and for paraffin, no tendency toward leveling off was observed. Rather, it was found that paraffin does not seem to be qualified as a suitable polymeric matrix to investigate phosphorus-based flame retardants in polyolefinic polymers.

**Figure 9:**
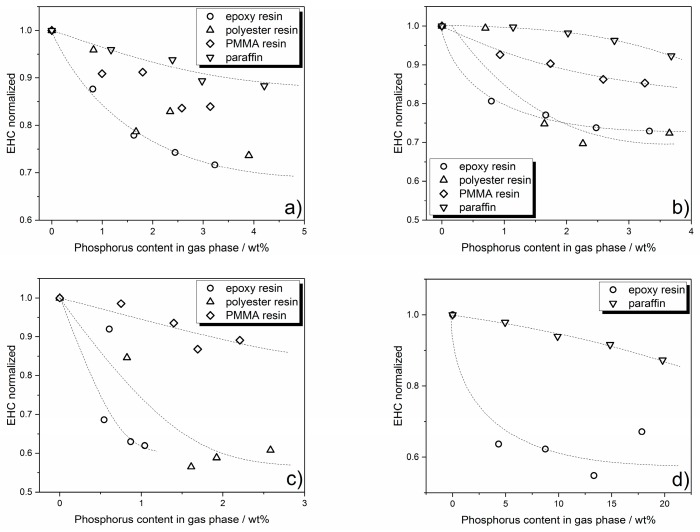
Effective heat of combustion versus the phosphorus content in the gas phase for the different polymeric systems flame retarded with: Exolit OP935 (**a**); Exolit OP1230 (**b**); BDP (**c**); and red phosphorus (**d**). Dotted lines are included as a guide to the eyes.

Additionally, the effective heat of combustion depending on phosphorus content in the gas phase was checked by comparing the different polymeric matrices flame retarded with the same flame retardant. This enables to make a statement about which flame retardant is most effective in which system. Since all systems exhibit different initial values for the non-flame retarded equivalent, the EHC had to be normalized in order to compare the effectiveness. The results of the comparisons are shown in Figure 9.

In order to compare the differences occurring with varying particle size distributions, both AlPi flame retardants Exolit OP935 and Exolit OP1230 are compared in the four different polymer matrices. Exolit OP935 is least efficient in paraffin, achieving only a reduction in EHC to 90%. In PMMA, a reduction to 85% is observed, followed by a reduction to around 75% in polyester resin. Exolit OP935 performs best in epoxy resin, when it comes to reduction in EHC.

Exolit OP1230 shows only a minor effect in paraffin, reducing the EHC to 95% at the highest loading. In PMMA resin, the efficiency is higher. The EHC is reduced up to 85%. In epoxy resin and polyester resin, Exolit OP1230 exhibits equally good EHC reduction, making both the ideal matrix for this flame retardant.

This comparison showed that there are slight differences in effectiveness depending on the polymer matrix resulting from different particle size distributions of Exolit OP935 and Exolit OP1230. Due to incorporation of BDP into the polymeric systems (Figure 9c), the EHC was reduced to around 60% in the epoxy resin at around 1 wt % of phosphorus in the gas phase. In the polyester resin, a higher phosphorus content in the gas phase was observed, reducing the EHC to around 55% at 1.5 wt % of released phosphorus. In the PMMA resin, only a reduction to around 85% was achieved.

Red phosphorus was incorporated into epoxy resin and paraffin. Both systems exhibit a totally different behavior. As shown in Figure 6, the EHC of the separately burning red phosphorus is lower than the EHC of the paraffin matrix. The mean EHC depicted here is decreasing with higher flame retardant load, because the duration of burning red phosphorus increases. It is not a result of an active flame inhibiting effect. In the epoxy system, red phosphorus shows a maximum reduction of EHC to around 55% while releasing 13 wt % of phosphorus into the gas phase.

This matrix comparison for each flame retardant showed, that the effectiveness with increasing flame retardant load and thus phosphorus content is highly dependent on the polymer matrix. All systems, except for paraffin, exhibit a more or less distinctive non-linear leveling off behavior. In addition, modifications such as the particle size distributions may play a minor role in whether a flame retardant is more effective in one or another polymeric matrix. Furthermore, paraffin was shown to be unsuitable to simulate the fire behavior of a polyolefinic polymer matrix.

#### 3.3.3. Dependency of Condensed Phase Activity on Phosphorus Content

**Figure 10:**
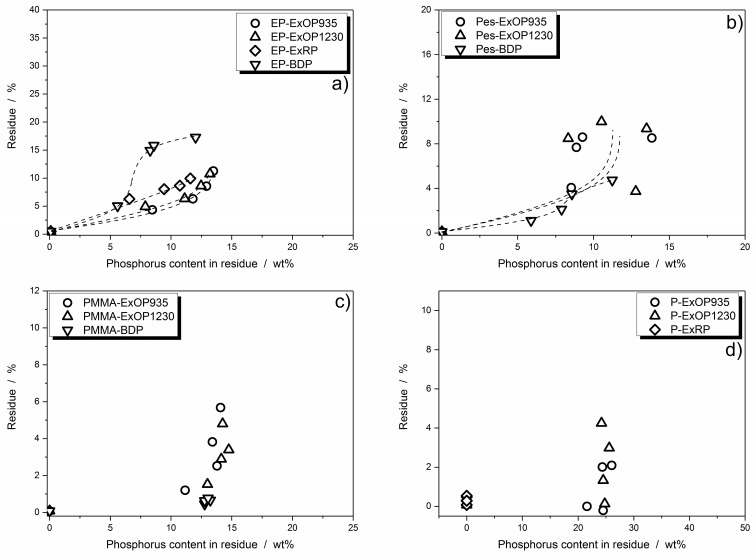
Residue plotted against the phosphorus content in the residue: DGEBA/IPDA (**a**); polyester (**b**); PMMA (**c**); and paraffin (**d**) system.

Phosphorus remaining in the residue after burning reacted during pyrolysis to contribute to condensed phase activity. The residue at the end of the cone calorimeter measurement is plotted versus the phosphorus content remaining in the residue in Figure 10. This plot gives information about the influence of increasing phosphorus content in the residue on residue formation.

**Figure 11:**
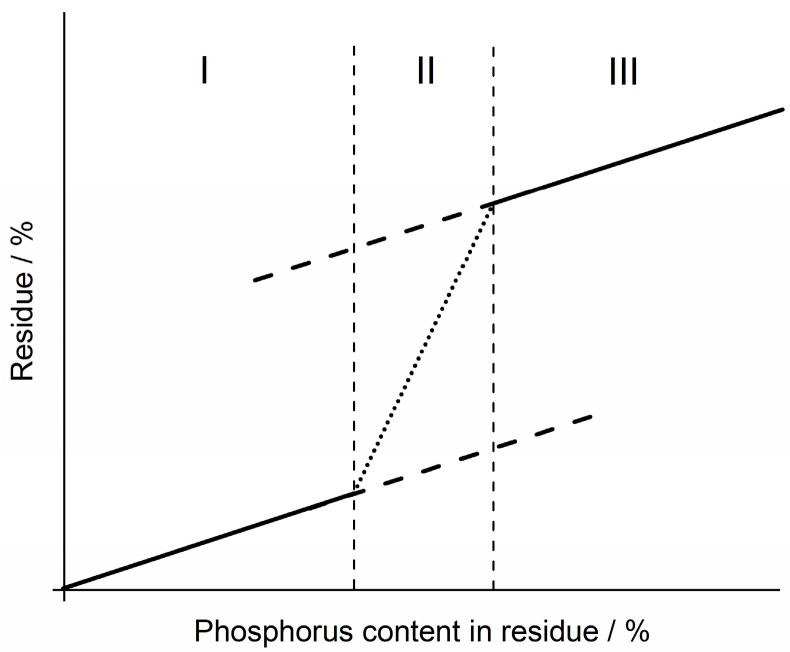
Proposed progression of residue formation with increasing phosphorus in the residue. The curve is divided into: an approximately linear phosphorus-residue relation (I); an area of inflection of the curve (II); and a continuous increase of residue with phosphorus content after the inflection point (III) [45].

We propose that the curves resulting from all of the measured formulations take S-shapes, or are assumed to take an S-shape, as depicted in Figure 11. This is because the buildup of a protective layer is ever more significant with increasing amounts of flame retardant, in this case phosphorus content. The point of inflection of the sigmoidal curves, described by area II in Figure 11, is considered to be the amount of phosphorus needed to induce formation of a protective layer. After this inflection point, the amount of residue flattens again and results in a continuous increase with increasing phosphorus content (Figure 11 III).

In epoxy resin, the curve of residue increase for the BDP formulations has its point of inflection at a relatively low phosphorus concentration in the residue. This means that a protective layer effect, and thus the level of decomposition, started to influence the burning behavior after the inflection point, found at around 7 wt % of phosphorus in the residue. This is in accordance with previous results, in which BDP was shown to be a good precursor for condensed phase activity [6]. Depending on the step height of the sigmoidal curve, the degree of decomposition changes, up to a point, where the material is incompletely pyrolyzed [44,45,46]. For BDP in epoxy resin, this step height is observed from around 5 wt % to 15 wt % of residue. For Exolit OP935 and OP1230, the highest concentration of phosphorus in the investigated samples was at around 13 wt %. Up to this point, it is observed that the slope of the curve gradually increases. It is imaginable, that with higher flame retardant load, and thus higher concentrations of phosphorus found in the residue, the curve will exhibit a point of inflection as well, and continue as a sigmoidal curve. Red phosphorus incorporated in epoxy resin released more than 90% of phosphorus into the gas phase during burning. Nevertheless, the concentration of phosphorus in the residue increased with higher flame retardant load. Since this increase was not as significant as for the AlPi flame retardants, the point of inflection will occur at a much higher concentration of phosphorus in the residue (Figure 10a).

BDP in the polyester resin had a similar effect as in the epoxy resin, exhibiting the sigmoidal curve progression at relatively low concentrations of phosphorus in the residue. However, the amount of residue was relatively low as well, with only 4 wt % at the highest phosphorus concentration measured. The area of inflection in this system lies at around 7 wt % to 9 wt % of phosphorus in the residue. Since the height of the curve increases only slightly during the inflection, by around 2%, the change in decomposition level was much less than in the epoxy resin system. Residue formation was much higher for Exolit OP935 and OP1230, but the amount of phosphorus remaining in the residue varied moderately, as was the case for the amount of phosphorus released into the gas phase (Figure 10b).

In the PMMA resin, BDP formed almost no residue at all. Only 1 wt % was built up at around 13 wt % of phosphorus in the residue. For Exolit OP 935 and OP1230 a residue formation of 5 wt % was observed at a phosphorus concentration of around 14 wt % in the residue, which was the highest flame retardant load investigated (Figure 10c). In paraffin, the AlPi flame retardants Exolit OP935 and OP1230 exhibited a very high concentration of phosphorus in the residue. The phosphorus concentration stagnated at around 24 wt %, while the amount of residue formation increased up to 4 wt %. Formulations with red phosphorus did not form any residue from which a sample could be taken for elemental analysis. Thus, it was assumed that all of the phosphorus content was released into the gas phase (Figure 10d). In the PMMA resin and paraffin system, a trend such as the one proposed in Figure 11 was not recognizable. In fact, the phosphorus concentration in the residue was observed to be constant in the range of 0 to 20 wt % of flame retardant load. However, it is conceivable, that with even higher load, the amount of phosphorus in the residue will increase additionally to the amount of residue.

For the epoxy and polyester resin formulations, it was observed that with increasing flame retardant load, the amount of phosphorus remaining in the residue increased as well, up to a point where the concentration of phosphorus stagnated. Because of the increase in residue formation, it was assumed that this is the point where the effect of a change in decomposition level, up to the presence of incompletely pyrolyzed polymer due to a protective layer effect contributes more and more to the overall flame retardancy performance.

**Figure 12:**
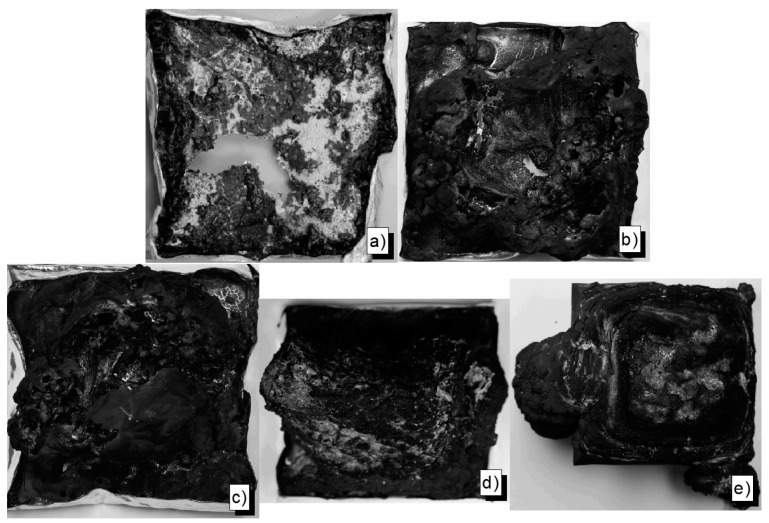
Residue pictures: EP (**a**); EP-15-ExOP935 (**b**); EP-15-ExOP1230 (**c**); EP-15-ExRP (**d**); and EP-25-BDP (**e**).

Residue pictures of flame retarded epoxy resin formulations reveal the influence of a protective layer on burning performance (Figure 12). Whereas residues from EP-15-ExOP935 and EP-15-ExOP1230 show beginnings of a closed residue surface that can serve as a protective layer, the residue of 15 wt % of RP in the epoxy resin is loose and brittle with no significant sign of a protective layer. In contrast to those examples, the completely closed surface of the residue of EP-25-BDP is evidence of a protective layer effect on burning performance, which prevented the complete combustion of some of the material.

**Figure 13:**
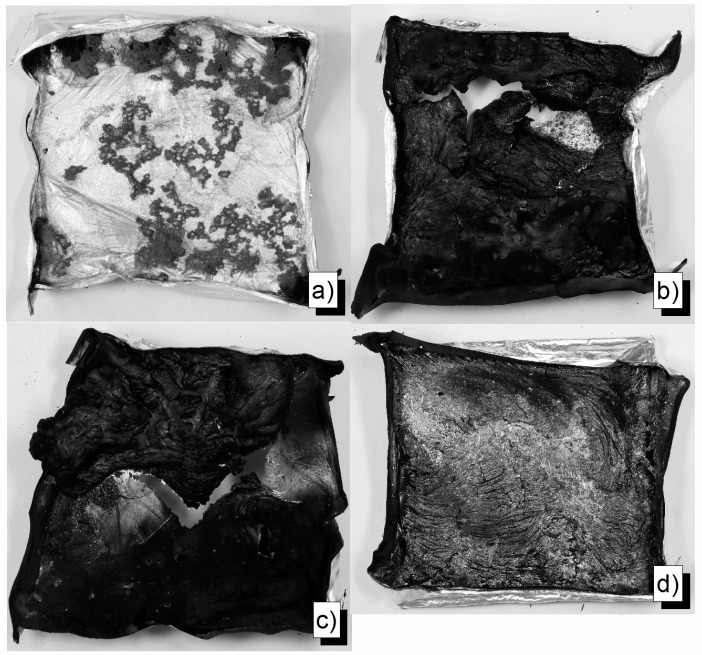
Residue pictures: Pes (**a**); Pes-15-ExOP935 (**b**); Pes-15-ExOP1230 (**c**); and Pes-25-BDP (**d**).

Exolit OP935 and OP1230 in polyester resin produced a residue that was very compact and had a closed surface, even though it was very light (Figure 13). However, due to the strong deformation during burning, the residue broke and a significant effect of a protective layer was lost. Pes-25-BDP, on the other hand, did not produce significant residue, but only a small, but compact and shiny char of 3.5 wt %. This demonstrates that the main flame retardancy effect has to come from gas phase activity, namely flame inhibition. In the next segment, this effect is quantified, along with charring and protection layer effects.

### 3.4. Quantification of Cone Calorimeter Results

Flame retardancy effects have been quantified for various flame retarded polymeric materials in previous works [46,47]. Equations (1) and (2) were used to calculate the theoretical values for PHRR and THE.

(1) $$HRR∼\chi \cdot (1-\mu )\cdot h_{c}^{0}$$

(2) $$THE∼\chi \cdot (1-\mu )\cdot h_{c}^{0}\cdot m_{0}$$

In these equations, χ is the combustion efficiency, $h_{c}^{0}$ is the heat of complete combustion of the fuel gases, *μ* is the char yield and *m*_0_ the mass of the specimen. The main premise is that the reduction of the effective heat of combustion $\chi \cdot h_{c}^{0}$ is influenced by flame inhibition, and this is displayed along with the effect of fuel reduction (1 − *μ*) and the altered density due to addition of a flame retardant. By measuring the formed residue as well as the reduction of EHC and the change in sample weight, a value for THE can be calculated. Comparing this calculated THE with the measured THE almost always shows perfect accordance. The values of measured and calculated reduction are expressed as percentages in Table 4, in which the non-flame retarded material is always set to 100%. Together with the effect of a protection layer, all three modes of action are displayed in the reduction of PHRR. The reduction in PHRR is calculated using Equation (1); however, this does not include the protective layer effect. Thus, the difference between calculated and measured PHRR reduction is a quantitative measure for a protective layer effect. To provide a better understanding of the approach, the calculation of the percentage of protective layer effect in EP-35-BDP is presented. The char yield of EP-35-BDP amounted to 17.3 wt %. Since the pure epoxy resin produced 0.5 wt % of char, the effective char yield resulting from addition of 35 wt % of BDP amounts to 16.9%. This equates to a fuel reduction (1 − *μ*) to 83.1% of the fuel released from pure epoxy resin. The effective heat of combustion $\chi \cdot h_{c}^{0}$ was measured in the cone calorimeter and was reduced to 62.0% of the EHC from pure epoxy resin. Multiplication of both values (0.831 × 0.620) leads to a calculated PHRR of 51.5% of the PHRR from pure epoxy resin. However, the measured PHRR was 33.8% of the PHRR from pure epoxy resin, which means that this further reduction has to be the result of other effects than fuel reduction and flame inhibition, which is mainly a protective layer effect. While more than those indices play a role in reducing the PHRR, it is considered to be one approximation to monitor protective layer effect. The change in steady state burning phase would be more accurate, but hardly any of our materials exhibited such a phase. The efficiency of a protective layer is also different, when the measurement method changes, leading to deviant behaviors in the cone calorimeter, UL94 and LOI tests.

For most of the specimens investigated, the effect of a protective layer was quite low. In the case of BDP in epoxy resin, the share of flame retardancy performance coming from a protective layer mode of action increased with increasing BDP load. This confirms the previous conclusions and complies with the conclusions from observing the residue pictures. Both AlPi flame retardants gave similar results in the epoxy resin, reducing the PHRR to around 45% at a flame retardant load of 20 wt %. They worked mainly in the gas phase. Red phosphorus, at a load of 20 wt %, was calculated to have the lowest protective layer effect of all tested flame retardants in epoxy resin, while achieving a satisfactory reduction in PHRR.

The ratio between PHRR reduction and the share in effectiveness that does not come from a protective layer effect was especially good in the polyester resin-BDP system. The PHRR was reduced to around 53%, and only 9% of this reduction is due to a protective layer effect. Twenty weight percent of ExOP1230 showed a slightly better PHRR reduction than ExOP935, but the share of the protective layer effect was also higher, at 39% compared to 26%.

BDP incorporated in the PMMA resin had the worst performance of any BDP formulation. At a load of 20 wt %, the PHRR was reduced to only 88%. Practically no protective layer effect could be detected. Both AlPi flame retardants showed about the same PHRR reduction at 20 wt % load, and the share in protection layer effect was similar as well.

In paraffin, a reduction of PHRR of around 64% was achieved with 20 wt % of Exolit OP935. However, 59% of the flame retardant effect was due to a protective layer. Those calculated values are not in accordance with the observations made during burning. No protective layer was formed during the forced flaming combustion tests in the cone calorimeter. Rather, the residue formed right before the flames extinguished.

According to the results depicted in Table 4, BDP and red phosphorus are the most effective flame retardants in epoxy resin. Up to a load of 25 wt %, the flame inhibition efficiency of BDP reaches its maximum. For red phosphorus, 15 wt % were concluded to be the optimal concentration in this system. In the polyester resin, Exolit OP1230 at a load of 10 to 20 wt % was most efficient. In the PMMA resin, the AlPi flame retardants showed their best performance at 20 wt %, while the incorporation of BDP did not result in satisfactory results even at high loads of 35 wt %. Differences in both tested AlPi flame retardants were most pronounced in paraffin. Twenty weight percent of Exolit OP935 in paraffin was concluded to be the best combination.

## 4. Conclusions

The use of easily preparable thermosets for investigating flame retardant modes of action, particularly flame inhibition in this work, was shown to be an effective method. Commercially available resins as well as paraffin were used to replace the time-consuming extrusion and injection molding of specimens. Although some flame retardants could not be implemented in certain matrices due to interferences with the curing process, much was learned from thermogravimetric analysis, infrared spectroscopy and burning behavior under forced flaming conditions. The dependence of EHC reduction and residue formation on phosphorus content in the gas phase and on residue, respectively, was revealed and investigated for each polymer matrix. Additionally, the performance in terms of EHC of a single flame retardant was compared among the different polymeric matrices. It was found that paraffin is not a suitable matrix for the rapid incorporation and investigation of modes of action of flame retardants and failed as a model for polyolefinic systems. The other polymer matrices gave great insight into the leveling off of flame inhibition efficiency of phosphorus-based flame retardants. Furthermore, the effect of particle size distribution was observed by comparing Exolit OP935 and Exolit OP1230 in epoxy resin and polyester resin, respectively. It was found that the finer grained Exolit OP935 is most effective in epoxy resin, whereas incorporation of Exolit OP1230 does not exhibit a large difference in epoxy resin and polyester resin. To investigate and explain the behavior of residue formation with increasing phosphorus content in the residue, a thesis for a crucial phosphorus concentration inducing a protective layer effect was proposed and proven in certain formulations, such as for BDP in epoxy or polyester resin. It was proposed that, for these specific formulations, there is a critical phosphorus concentration, at which the level of degradation changes significantly, up to the retention of incompletely pyrolyzed polymer due to a protective layer. The results were quantified with simple calculations and it was shown that they are in great accordance with observed and measured outcomes. However, it has to be mentioned that flame retardant behavior and performance in a polymeric system is not easy to generalize. There are noticeable trends when it comes to the changes in effectiveness and flame retardant mode of action of varying loads of BDP in epoxy resin, for example, but they do not allow predictions of behavior for a different epoxy-based polymeric system. These systems do not give a statement about the fire behavior of any epoxy-, acrylate- or polyester-based polymer, besides the ones studied. Overall, several new relations between polymer matrix, phosphorus species, phosphorus concentration and results under forced flaming conditions were clarified.

## Figures and Tables

**Table 1:** Nomenclature of the produced flame retarded polymeric materials with *x* = concentration of the flame retardant, 5, 10, 15 or 20 wt % for Exolit OP935, OP1230 and RP607 and 10, 20, 25 and 35 wt % for BDP respectively.

	Exolit OP935	Exolit OP1230	Exolit RP607	BDP
**DGEBA/IPDA**	EP-*x*-ExOP935	EP-*x*-ExOP1230	EP-*x*-ExRP	EP-*x*-BDP
**PMMA-resin**	PMMA-*x*-ExOP935	PMMA-*x*-ExOP1230	---	PMMA-*x*-BDP
**Polyester-resin**	Pes-*x*-ExOP935	Pes-*x*-ExOP1230	---	Pes-*x*-BDP
**Paraffin**	P-*x*-ExOP935	P-*x*-ExOP1230	P-*x*-ExRP	---

**Table 2:** Results from thermogravimetric analysis (TGA). T_5% ML_ is the temperature at which 5 wt % of the mass is lost, T_DTGmax_ is the temperature of the maximum of the mass loss rate. The residue was determined at 800 °C.

Material	T_5% ML_	T_DTGmax_	Residue
	°C	°C	wt %
**EP**	326 ± 1	360 ± 2	10.7 ± 0.1
**EP-10-ExOP935**	320 ± 1	355 ± 1	12.1 ± 0.3
**EP-10-ExOP1230**	320 ± 2	353 ± 1	12.1 ± 0.3
**EP-5-ExRP**	318 ± 1	349 ± 1	17.0 ± 0.2
**EP-10-BDP**	265 ± 1	365 ± 1	3.0 ± 0.1
**Pes**	204 ± 1	380 ± 2	2.9 ± 0.1
**Pes-10-ExOP935**	211 ± 1	378 ± 1	14.0 ± 0.4
**Pes-10-ExOP1230**	210 ± 1	382 ± 2	11.7 ± 0.2
**Pes-20-BDP**	277 ± 2	380 ± 2	2.6 ± 0.1
**PMMA**	192 ± 1	370 ± 2	1.7 ± 0.1
**PMMA-10-ExOP935**	229 ± 2	368 ± 1	4.6 ± 0.2
**PMMA-10-ExOP1230**	228 ± 1	370 ± 2	4.5 ± 0.1
**PMMA-10-BDP**	246 ± 2	364 ± 1	0.7 ± 0.1
**P**	223 ± 1	296 ± 1	0.1 ± 0.1
**P-10-ExOP935**	207 ± 1	274 ± 1	0.2 ± 0.1
**P-10-ExOP1230**	225 ± 2	454 ± 3	1.4 ± 0.2
**P-10-ExRP**	207 ± 1	273 ± 1	2.1 ± 0.2

**Table 3:** Cone calorimeter results.

System	t_ig_	PHRR	THE	EHC	Residue at End of Test
	s	kW/m^2^	MJ/m^2^	MJ/kg	wt %
**EP**	39 ± 1	1858 ± 41	81.3 ± 0.1	24.7 ± 0.1	0.5 ± 0.0
**EP-5-ExOP935**	30 ± 1	1632 ± 4	71.1 ± 2.1	21.6 ± 0.4	4.3 ± 0.3
**EP-10-ExOP935**	32 ± 1	969 ± 57	59.0 ± 1.3	19.2 ± 0.3	6.3 ± 0.3
**EP-15-ExOP935**	30 ± 1	864 ± 9	57.0 ± 0.3	18.3 ± 0.0	8.6 ± 0.3
**EP-20-ExOP935**	30 ± 0	817 ± 25	52.3 ± 0.1	17.7 ± 0.1	11.3 ± 0.6
**EP-5-ExOP1230**	31 ± 2	1105 ± 52	71.6 ± 0.3	19.9 ± 1.4	4.9 ± 0.1
**EP-10-ExOP1230**	32 ± 1	1097 ± 50	61.1 ± 0.1	19.0 ± 0.0	6.3 ± 0.2
**EP-15-ExOP1230**	30 ± 0	1007 ± 16	58.9 ± 0.8	18.2 ± 0.2	8.6 ± 0.2
**EP-20-ExOP1230**	31 ± 2	861 ± 2	54.0 ± 0.3	18.0 ± 0.1	10.8 ± 0.0
**EP-5-ExRP**	29 ± 1	964 ± 16	51.1 ± 1.9	15.7 ± 0.3	6.3 ± 0.1
**EP-10-ExRP**	33 ± 1	786 ± 24	47.2 ± 1.0	15.4 ± 0.4	8.0 ± 0.1
**EP-15-ExRP**	34 ± 2	673 ± 125	42.4 ± 0.9	13.5 ± 0.4	8.7 ± 0.5
**EP-20-ExRP**	32 ± 2	873 ± 56	50.1 ± 0.4	16.6 ± 0.3	9.9 ± 1.5
**EP-10-BDP**	25 ± 3	1468 ± 44	74.0 ± 4.1	22.7 ± 1.4	5.0 ± 0.3
**EP-20-BDP**	26 ± 1	688 ± 3	49.0 ± 0.7	16.9 ± 0.1	14.9 ± 1.1
**EP-25-BDP**	22 ± 2	660 ± 1	45.0 ± 0.3	15.5 ± 0.1	15.8 ± 1.7
**EP-35-BDP**	28 ± 1	628 ± 3	42.5 ± 0.6	15.3 ± 0.3	17.3 ± 0.4
**Pes**	26 ± 1	1017 ± 16	94.3 ± 2.9	19.9 ± 0.9	0.1 ± 0.1
**Pes-5-ExOP935**	33 ± 2	854 ± 38	85.5 ± 0.4	19.1 ± 0.1	4.1 ± 0.2
**Pes-10-ExOP935**	32 ± 1	599 ± 6	64.5 ± 1.3	15.7 ± 0.1	7.7 ± 0.5
**Pes-15-ExOP935**	36 ± 2	577 ± 29	70.9 ± 0.0	16.5 ± 0.2	8.5 ± 0.3
**Pes-20-ExOP935**	34 ± 1	521 ± 15	60.9 ± 3.2	14.7 ± 0.6	8.6 ± 0.5
**Pes-5-ExOP1230**	31 ± 2	969 ± 33	89.8 ± 0.1	19.8 ± 0.1	3.7 ± 0.3
**Pes-10-ExOP1230**	32 ± 5	428 ± 27	69.4 ± 2.2	14.9 ± 2.0	8.5 ± 0.1
**Pes-15-ExOP1230**	30 ± 2	603 ± 37	73.4 ± 4.0	13.9 ± 2.3	9.4 ± 0.4
**Pes-20-ExOP1230**	33 ± 1	456 ± 8	61.2 ± 1.7	14.4 ± 0.4	10.0 ± 0.1
**Pes-10-BDP**	35 ± 1	727 ± 80	77.8 ± 1.4	16.9 ± 0.3	1.1 ± 0.4
**Pes-20-BDP**	32 ± 1	629 ± 63	66.5 ± 0.7	11.3 ± 3.2	2.1 ± 0.5
**Pes-25-BDP**	37 ± 6	570 ± 17	59.6 ± 0.6	11.7 ± 1.7	3.5 ± 0.5
**Pes-35-BDP**	34 ± 5	538 ± 49	55.9 ± 1.4	12.1 ± 0.4	4.8 ± 0.3
**PMMA**	32 ± 1	1051 ± 41	106.9 ± 0.6	25.1 ± 0.5	0.1 ± 0.0
**PMMA-5-ExOP935**	26 ± 1	836 ± 1	93.7 ± 2.5	22.8 ± 0.7	1.2 ± 0.2
**PMMA-10-ExOP935**	25 ± 1	792 ± 21	88.8 ± 0.5	22.8 ± 0.2	2.5 ± 0.1
**PMMA-15-ExOP935**	24 ± 1	740 ± 37	83.8 ± 0.3	20.9 ± 0.1	3.8 ± 0.0
**PMMA-20-ExOP935**	22 ± 1	615 ± 33	81.8 ± 1.0	21.0 ± 0.4	5.7 ± 0.5
**PMMA-5-ExOP1230**	23 ± 1	852 ± 20	97.0 ± 1.0	23.2 ± 1.8	1.5 ± 0.1
**PMMA-10-ExOP1230**	22 ± 0	722 ± 16	89.2 ± 1.1	22.6 ± 0.4	2.9 ± 0.0
**PMMA-15-ExOP1230**	24 ± 2	678 ± 63	86.6 ± 3.0	21.6 ± 0.6	3.4 ± 0.1
**PMMA-20-ExOP1230**	26 ± 2	620 ± 4	82.8 ± 0.6	21.4 ± 04	4.8 ± 0.5
**PMMA-10-BDP**	29 ± 0	1079 ± 25	108.4 ± 0.3	24.7 ± 2.3	0.5 ± 0.1
**PMMA-20-BDP**	31 ± 1	994 ± 38	97.4 ± 0.9	23.4 ± 0.4	0.6 ± 0.0
**PMMA-25-BDP**	28 ± 1	868 ± 48	91.5 ± 1.9	21.7 ± 0.1	0.7 ± 0.1
**PMMA-35-BDP**	28 ± 1	928 ± 19	93.0 ± 0.2	22.3 ± 0.4	0.8 ± 0.1
**P**	40 ± 3	2996 ± 24	210.1 ± 2.9	44.1 ± 0.5	0.1 ± 0.0
**P-5-ExOP935**	32 ± 1	1935 ± 167	198.8 ± 7.1	42.3 ± 0.7	0.0 ± 0.0
**P-10-ExOP935**	27 ± 2	1593 ± 96	189.9 ± 27.1	41.4 ± 1.0	0.0 ± 0.2
**P-15-ExOP935**	23 ± 1	1371 ± 101	183.8 ± 1.3	39.4 ± 0.1	2.1 ± 0.3
**P-20-ExOP935**	23 ± 2	1130 ± 69	187.6 ± 3.7	38.9 ± 0.4	1 ± 0.6
**P-5-ExOP1230**	37 ± 2	2872 ± 318	197.2 ± 1.5	44.0 ± 0.2	0.1 ± 0.1
**P-10-ExOP1230**	42 ± 6	2868 ± 123	209.9 ± 2.9	43.3 ± 0.3	1.3 ± 0.8
**P-15-ExOP1230**	29 ± 1	2559 ± 223	195.8 ± 3.5	42.4 ± 0.7	2.9 ± 0.3
**P-20-ExOP1230**	27 ± 4	2257 ± 67	188.6 ± 12.6	40.7 ± 1.6	4.3 ± 1.0
**P-5-ExRP**	33 ± 1	2945 ± 13	212.5 ± 10.9	43.2 ± 0.2	0.5 ± 0.5
**P-10-ExRP**	34 ± 1	2781 ± 150	201.1 ± 1.6	41.4 ± 0.1	0.5 ± 0.0
**P-15-ExRP**	29 ± 3	2559 ± 219	191.8 ± 1.5	40.4 ± 0.6	0.2 ± 0.1
**P-20-ExRP**	35 ± 4	2519 ± 35	189.9 ± 2.6	38.5 ± 0.7	0.3 ± 0.6

**Table 4:** Quantification of the reduction in THE and PHRR due to the flame retardancy modes of action: charring, gas phase action, and residual protection layer for all of the examined systems.

Material	(1 − µ)	$\chi \cdot h_{c}^{0}$	m_0_	Cal. THE	THE	Cal. PHRR	PHRR	Prot. Layer
	%	%	%	%	%	%	%	%
**EP**	100.0	100.0	100.0	100.0	100.0	100.0	100.0	0.0
**EP-5-ExOP935**	96.1	87.7	103.8	87.5	87.5	84.3	87.9	−4.3
**EP-10-ExOP935**	94.2	77.9	98.9	72.5	72.6	73.3	52.2	28.9
**EP-15-ExOP935**	91.9	74.3	102.7	70.1	70.1	68.3	46.5	31.9
**EP-20-ExOP935**	89.2	71.7	100.7	64.4	64.4	63.9	44.0	31.2
**EP-5-ExOP1230**	95.6	80.6	105.5	81.3	88.2	77.1	59.5	22.8
**EP-10-ExOP1230**	94.1	77.1	103.6	75.2	75.2	72.5	59.0	18.6
**EP-15-ExOP1230**	91.8	73.8	107.0	72.5	72.5	67.8	54.2	20.0
**EP-20-ExOP1230**	89.7	72.9	101.7	66.5	66.5	65.4	46.3	29.2
**EP-5-ExRP**	94.2	63.7	104.8	62.9	62.9	60.0	51.9	13.5
**EP-10-ExRP**	92.4	62.3	101.0	58.1	58.1	57.5	42.3	26.5
**EP-15-ExRP**	91.8	54.8	103.7	52.2	52.1	50.3	36.2	27.9
**EP-20-ExRP**	90.5	67.2	101.4	61.7	61.6	60.8	47.0	22.7
**EP-10-BDP**	95.4	92.0	103.7	91.1	91.0	87.8	79.0	10.0
**EP-20-BDP**	85.5	68.6	102.8	60.3	60.3	58.7	37.0	36.9
**EP-25-BDP**	84.6	63.0	104.0	55.4	55.4	53.3	35.5	33.3
**EP-35-BDP**	83.1	62.0	101.4	52.3	52.3	51.5	33.8	34.4
**Pes**	100.0	100.0	100.0	100.0	100.0	100.0	100.0	0.0
**Pes-5-ExOP935**	96.0	95.9	101.3	93.3	90.7	92.1	84.0	8.8
**Pes-10-ExOP935**	92.4	78.7	96.6	70.2	68.4	72.7	58.9	18.9
**Pes-15-ExOP935**	91.6	82.9	102.3	77.6	75.2	75.9	56.8	25.2
**Pes-20-ExOP935**	91.5	73.6	98.9	66.6	64.6	67.4	51.2	24.0
**Pes-5-ExOP1230**	96.4	99.5	102.4	98.2	95.2	95.9	95.3	0.5
**Pes-10-ExOP1230**	91.6	74.8	102.4	70.2	73.7	68.6	42.1	38.6
**Pes-15-ExOP1230**	90.8	69.7	101.1	63.9	77.8	63.2	59.3	6.2
**Pes-20-ExOP1230**	90.1	72.4	101.3	66.1	64.9	65.3	44.8	31.3
**Pes-10-BDP**	99.0	84.6	100.3	84.0	82.5	83.8	71.5	14.7
**Pes-20-BDP**	98.0	56.5	100.3	55.5	70.5	55.4	61.8	-11.7
**Pes-25-BDP**	96.6	58.9	99.3	56.5	63.2	56.9	56.0	1.4
**Pes-35-BDP**	95.4	60.8	98.9	57.3	59.3	58.0	52.9	8.8
**PMMA**	100.0	100.0	100.0	100.0	100.0	100.0	100.0	0.0
**PMMA-5-ExOP935**	98.9	90.9	97.5	87.6	87.6	89.9	79.6	11.4
**PMMA-10-ExOP935**	97.6	91.2	93.4	83.1	83.1	89.0	75.4	15.2
**PMMA-15-ExOP935**	96.3	83.6	97.4	78.4	78.4	80.5	70.4	12.6
**PMMA-20-ExOP935**	94.4	83.9	96.5	76.4	76.5	79.2	58.6	26.0
**PMMA-5-ExOP1230**	98.6	92.6	99.4	90.8	90.7	91.3	81.1	11.1
**PMMA-10-ExOP1230**	97.2	90.3	95.0	83.4	83.4	87.8	68.7	21.7
**PMMA-15-ExOP1230**	96.7	86.3	97.1	80.9	81.0	83.4	64.5	22.6
**PMMA-20-ExOP1230**	95.3	85.3	95.2	77.4	77.4	81.3	59.0	27.4
**PMMA-10-BDP**	99.6	104.6	97.6	101.7	101.4	104.2	102.6	1.5
**PMMA-20-BDP**	99.5	93.5	97.9	91.1	91.1	93.0	94.6	-1.7
**PMMA-25-BDP**	99.4	86.8	99.2	85.6	85.6	86.3	82.6	4.3
**PMMA-35-BDP**	99.3	89.1	98.3	87.0	87.0	88.5	88.3	0.2
**P**	100.0	100.0	100.0	100.0	100.0	100.0	100.0	0.0
**P-5-ExOP935**	100.1	95.9	98.2	94.2	94.7	96.0	64.6	32.7
**P-10-ExOP935**	100.1	93.2	96.3	90.4	90.4	93.9	53.2	43.4
**P-15-ExOP935**	98.0	89.4	99.1	86.8	87.5	87.5	45.8	47.7
**P-20-ExOP935**	98.1	88.3	103.3	89.5	89.3	86.6	37.7	56.5
**P-5-ExOP1230**	99.9	99.7	94.5	94.1	93.9	99.7	95.9	3.8
**P-10-ExOP1230**	98.7	98.2	102.9	99.8	99.9	97.0	95.7	1.3
**P-15-ExOP1230**	97.1	96.3	98.0	91.6	93.2	93.5	85.4	8.6
**P-20-ExOP1230**	95.8	92.3	102.2	90.4	89.8	88.5	75.3	14.9
**P-5-ExRP**	99.6	97.9	103.1	100.5	101.1	97.5	98.3	-0.8
**P-10-ExRP**	99.5	93.9	102.5	95.8	95.7	93.5	92.8	0.7
**P-15-ExRP**	99.8	100.7	100.1	91.5	91.3	91.4	85.4	6.5
**P-20-ExRP**	99.8	87.3	104.2	90.8	90.4	87.1	84.1	3.5
